# p53-R273H upregulates neuropilin-2 to promote cell mobility and tumor metastasis

**DOI:** 10.1038/cddis.2017.376

**Published:** 2017-08-10

**Authors:** Tao Lv, Xianqiang Wu, Lijuan Sun, Qingyong Hu, Yang Wan, Liang Wang, Zhiqiang Zhao, Xiao Tu, Zhi-Xiong Jim Xiao

**Affiliations:** 1 Center of Growth, Metabolism and Aging, and Key Laboratory of Bio-Resource and Eco-Environment, Ministry of Education, College of Life Sciences, Sichuan University, Chengdu 610065, China

## Abstract

Mounting evidence indicates that hotspot p53 mutant proteins often possess gain-of-function property in promoting cell mobility and tumor metastasis. However, the molecular mechanisms are not totally understood. In this study, we demonstrate that the hotspot mutation, p53-R273H, promotes cell migration, invasion *in vitro* and tumor metastasis *in vivo*. p53-R273H significantly represses expression of DLX2, a homeobox protein involved in cell proliferation and pattern formation. We show that p53-R273H-mediated DLX2 repression leads to upregulation of Neuropilin-2 (NRP2), a multifunctional co-receptor involved in tumor initiation, growth, survival and metastasis. p53-R273H-induced cell mobility is effectively suppressed by DLX2 expression. Furthermore, knockdown of NRP2 significantly inhibits p53-R273H-induced tumor metastasis in xenograft mouse model. Together, these results reveal an important role for DLX2-NRP2 in p53-R273H-induced cell mobility and tumor metastasis.

Tumor suppressor protein p53 is activated in response to various cellular stresses to transactivate a set of target genes involved in cell cycle arrest, apoptosis, senescence, DNA repair and cancer cell metabolism.^1^ It is well-documented that more than 50% of human tumors and cancers contain a mutation in p53 gene with the vast majority of these mutations occur in the DNA-binding domain.^2, 3^ The primary cancer-associated alterations in p53 gene are a single amino acid substitution.^4^ Several mutations clustered within the central DNA-binding domain are frequently found in cancers, refereed as ‘hotspot’ mutations, which include R175, G245, R248, R249, R273 and R282.^5^ Mutant p53 proteins usually have lost its DNA sequence-specific transcription function, they can also inhibit wild-type p53 function in a dominant negative fashion,^6^ since p53 protein usually exerts as a tetramer. It is now known that mutant p53 proteins not only lost their transcriptional function (loss of function), but also commonly obtain new function (gain of function).^7^ For instance, p53-R175H exhibits a gain of oncogenic function in driving cell migration and invasion.^4, 8^ Studies from animal models have confirmed that mice bearing a mutant p53 allele show more aggressive and metastatic tumor than p53 null.^9, 10, 11^ In human cancers, mutant p53 expression has been linked to poorer prognosis.^12^

Distal-less homeobox 2 (DLX2) plays an important role in control of cell migration,^13^ proliferation,^14^ differentiation,^15^ neurogenesis^16, 17^ and pattern formation.^18^ Downregulation of DLX expression has been reported in human solid and hematologic malignancies, and in lymphoblastic leukemias.^19, 20, 21^

Neuropilins (NRPs) are transmembrane surface glycoproteins consisting of two members, Neuropilin-1 (NRP1) and Neuropilin-2 (NRP2). NRP2 functions as a co-receptor of SEMA3, vascular endothelial growth factor and transforming growth factor *β* 1 and is important for cell proliferation, survival, epithelial-mesenchymal transition and metastasis.^22^ It has recently become widely acknowledged that upregulation of NRP2 expression is associated with tumorigenesis and, in particular, tumor metastasis.^23^

Here, we investigated the functions of mutant p53 protein (R273H, R175H, R248W) in regulation of cell migration and invasion. We demonstrated that p53-R273H upregulates NRP2 expression via suppression of DLX2 transcription, leading to increased cell mobility. In addition, knockdown of NRP2 significantly inhibits p53-R273H-induced tumor metastasis *in vivo*. Together, this study demonstrates the importance of DLX2-NRP2 pathway in p53-R273H-induced cell mobility, epithelial-mesenchymal transition and tumor metastasis.

## Results

### Expression of hotspot mutant p53 promotes scattering cell growth and accelerates cell migration

**Figure 1:**
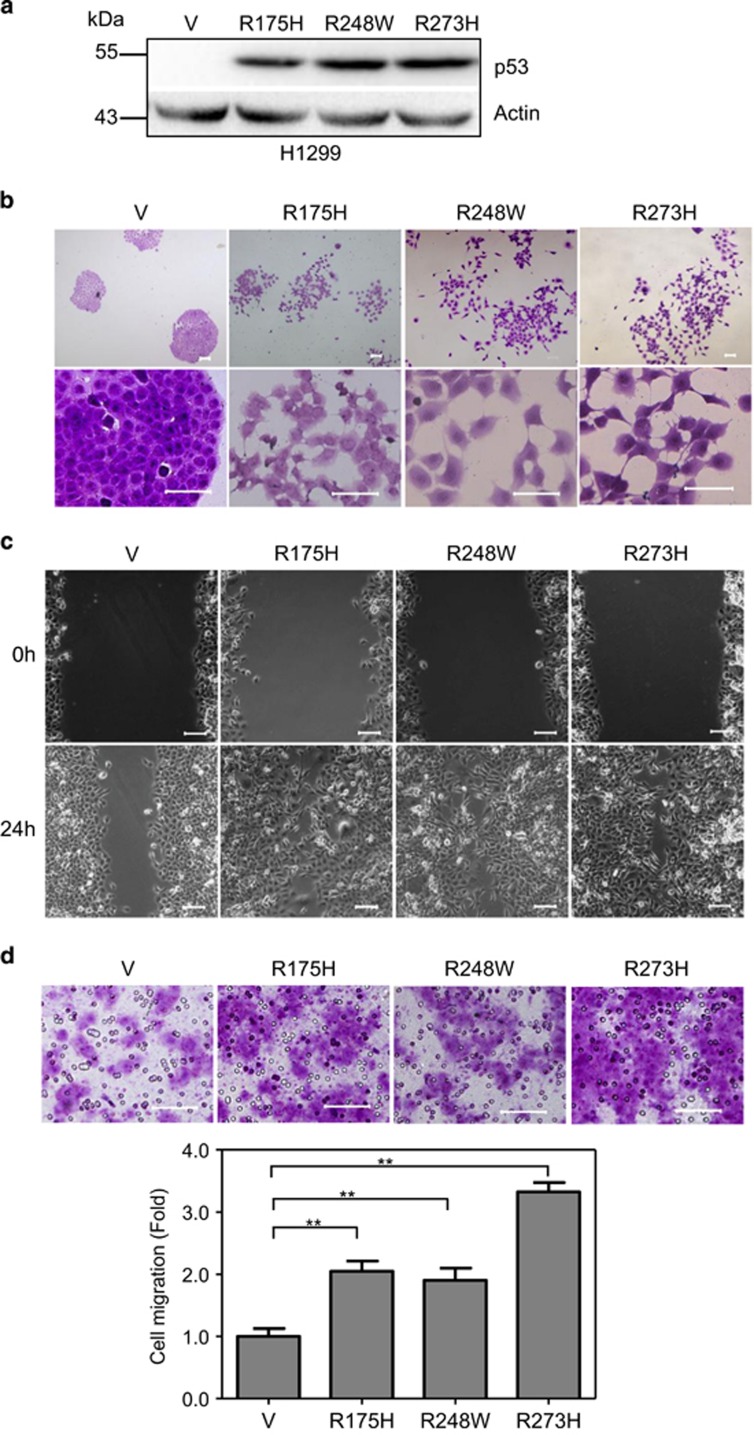
Expression of mutant p53 promotes cell morphological changes and cell migration. (**a**–**d**) H1299 cells stably expressing vector control (V) or a mutant p53 allele (p53-R175H, p53-R248W or p53-R273H) were subjected to western blot analyses using p53 (DO-1) antibody (**a**); subjected to photograph under a phase-contrast microscope (**b**); subjected to wound-healing assay (**c**); or subjected to transwell assays (**d**). Scale bar =100 *μ*m. ** indicated *P*<0.01. Results are presented as means and S.E. from three independent experiments in triplicates

It is well documented that hotspot mutations of p53 gene, in particular, R175H, R248W and R273H, are loss of function alleles as they are unable to transactivate downstream genes involved cell growth control and apoptosis.^24^ However, it becomes clear that these mutant alleles, in addition, exhibit gain of new functions in promoting tumorigenesis, including promoting cancer metastasis,^25^ yet the molecular mechanisms are not totally understood. To investigate the molecular mechanisms by which the hotspot p53 mutations promote cancer metastasis, we first examined the effects of these mutant p53 alleles on the ability of cell migration and invasion. As shown in Figure 1, while human non-small cell lung cancer H1299 cells, which do not express endogenous p53, grew into typical epithelial morphology exhibiting tightly packed colonies and formed a continuous sheet with no intercellular spaces, expression of either p53-R175H, p53-R248W or p53-R273H led to scattering cell growth with mobile cell morphologies showing lamellipodia on the leading edge of the cells (Figures 1a and b). In addition, expression of mutant p53 significantly promoted cell migration, as evidenced by wound healing assay (Figure 1c) and transwell assays (Figure 1d). Notably, cells expressing p53-R175H and p53-R248W migrated approximately two times faster than that of control cells as assessed by transwell assays. By contrast, cells expressing p53-R273H migrated approximately three times faster than that of control cells, although the protein expression levels were comparable (Figure 1a). Expression of mutant p53 in human colon cancer HCT116 p53^−/−^ cells also promoted scattering cell growth and accelerated cell migration (Supplementary Figures S1A–C). These observations indicate that hotspot p53 mutations promote scattering cell growth and accelerate cell migration.

To examine whether expression of mutant p53 is indeed responsible for cell morphological changes, scattering cell growth and accelerated cell migration, we used an shRNA specific for p53 to knock-down p53-R273H in H1299 cells. As shown in Supplementary Figures S2A and B, p53-R273H was effectively knocked-down, which led to almost complete reverse of scattering cell growth and mobile cell morphology induced by p53-R273H (Supplementary Figure S2C). In addition, knockdown of p53-R273H resulted in total reverse of cell migration and invasion compared to control cells (Supplementary Figures S2D–F). Together, these results indicate that p53-R273H is responsible for scattering cell growth, morphological changes and accelerated cell migration/invasion in H1299 cells.

### Downregulation of DLX2 promotes scattering cell growth, cell migration and invasion

Several studies have attempted to explore the molecular mechanisms by which mutant p53 exerts its function.^26^ It has been reported that p53-R175H can downregulate expression of DLX2,^27^ a homeobox protein important in control of cell migration.^13^ We therefore investigated whether DLX2 plays a role in p53-R273H- induced cell mobility.

**Figure 2:**
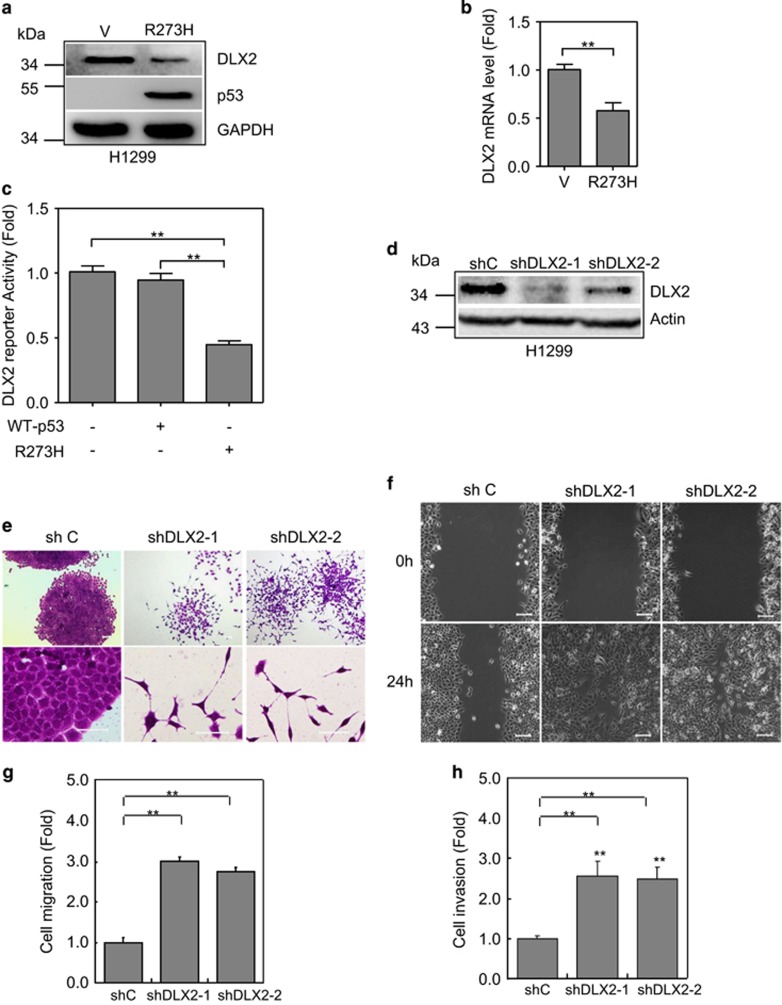
p53-R273H inhibits DLX2 expression in promoting cell scattering growth, migration and invasion. (**a**,**b**) Stable H1299 cells expressing p53-R273H (R273H) or a vector control (V) were subjected to western blot analyses for DLX2 expression (**a**) or Q-PCR analysis (**b**). Results are presented as means and S.E. from three independent experiments in triplicates. ** indicated *P*<0.01. (**c**) H1299 cells were co-transfected with DLX2-Lux, TK-Renilla, and a plasmid encoding either wild-type p53, p53-R273H or vector. Luciferase activities was normalized to Renilla activity and presented as fold of activation (mean±S.E.) from three independent experiments in triplicates. (**d**–**h**) Stable H1299 cells expressing shRNA specific for DLX2 (shDLX2-1, shDLX2-2) or GFP^8^ were subjected to western blot analyses (**d**). Cells were also subjected to photograph using microscopy (**e**); to wound-healing assay (**f**) and to transwell migration (**g**) or invasion (**h**) assays. Results are presented as means and S.E. from three independent experiments in triplicates. Scale bar =100 *μ*m. ** indicated *P*<0.01

As shown in Figures 2a and b, p53-R273H significantly downregulated DLX2 in H1299 cells, as assayed by western blot analyses and Q-PCR analyses. Furthermore, p53-R273H but not wild-type p53 downregulated DLX2 reporter activity (Figure 2c). Next, we examined the effects of knockdown of DLX2 on cell morphology and cell migration/invasion. Two specific DLX2 shRNAs were used to knockdown endogenous DLX2 in H1299 cells, respectively (Figure 2d). Knockdown of DLX2 dramatically induced scattering cell growth (Figure 2e), cell migration (Figures 2f and g) and invasion (Figure 2h). Taken together, these results indicate that R273H specifically downregulated DLX2 expression, and reduced DLX2 expression significantly promotes scattering cell growth, cell migration and invasion.

### DLX2 plays a critical role in p53-R273H-induced scattering cell growth, cell migration and invasion

**Figure 3:**
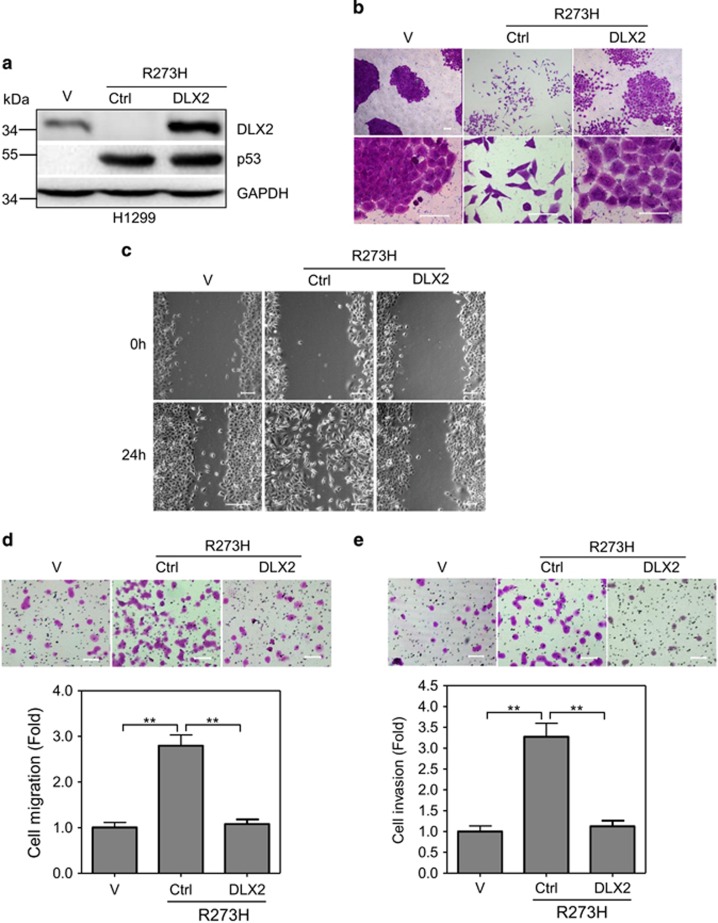
Ectopic expression of DLX2 in H1299 cells inhibits p53-R273H-induced scattering cell growth, migration and invasion. (**a**–**e**) H1299 cells stably expressing p53-R273H were infected with recombinant lentivirus encoding DLX2. Cells were subjected to western blot analyses (**a**) or photograph using microscopy (**b**); to wound-healing assay (**c**) and to transwell migration (**d**) or invasion (**e**) assays. Results are presented as means and S.E. from three independent experiments in triplicates. Scale bar =100 *μ*m. ** indicated *P*<0.01

Since expression of p53-R273H in H1299 induces scattering cell growth and cell migration, concomitant with decreased expression of DLX2, we therefore investigated whether downregulation of DLX2 is responsible for p53-R273H-mediated changes of cell motility. In the rescuing experiments, ectopic expression of DLX2 significantly, but not completely, inhibited p53-R273H-induced scattering cell growth (Figures 3a and b). However, ectopic expression of DLX2 completely inhibited cell migration and invasion induced by p53-R273H (Figures 3c–e). Taken together, these results demonstrate that p53-R273H promotes scattering cell growth, cell migration and invasion via downregulation of DLX2.

### p53-R273H promotes scattering cell growth, cell migration and invasion via upregulation of NRP2 expression

**Figure 4:**
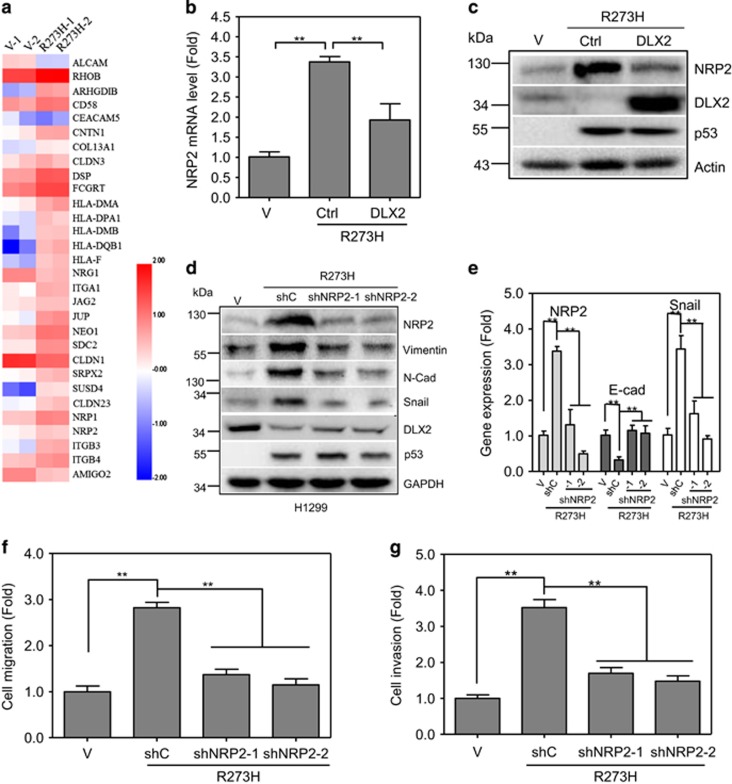
p53-R273H upregulates NRP2 to promote cell migration. (**a**) H1299 cells stably expressing mutant p53-R273H were subjected to RNA-seq based digital gene expression (DGE) analyses. Differentially expressed genes were identified by a *P*-value ⩽ 0.01 and an expression change of twofold or more between the two samples. (**b**,**c**) H1299 cells stably expressing p53-R273H were infected with recombinant lentivirus encoding DLX2. Cells were subjected to Q-PCR analysis (**b**) for NRP2 expression, and to western blot analyses (**c**) for NRP2, DLX2 and p53 expression. Results are presented as means and S.E. from three independent experiments in triplicates. ** indicated *P*<0.01. (**d**–**g**) Stable H1299 cells expressing p53-R273H were infected with recombinant lentivirus encoding a shRNA specific for NRP2 (shNRP2-1 or shNRP2-2) or a shGFP control were subjected to western blot analyses, as indicated (**d**); or to Q-PCR experiments to measure expression of NRP2, E-cadherin or Snail, as shown (**e**); or to transwell migration (**f**) or invasion (**g**) assays. Results are presented as means and S.E. from three independent experiments in triplicates. Scale bar =100 *μ*m. ** indicated *P*<0.01

It has been shown that DLX2 functions as a transcription factor to regulate a set of genes involved in variety of different biological processes.^18^ Notably, microarrays analyses showed that NRP2, a co-receptor of several membrane receptors including transforming growth factor *β*1, PDGF, HGF and FGF receptors,^28^ is a direct downstream target that is inhibited by DLX2.^13^ Consistent with previous report, our RNA-Seq analyses also indicated that expression of p53-R273H in H1299 cells significantly upregulated NRP2 (Figure 4a). Indeed, knockdown of DLX2 significantly upregulated NRP2 expression in H1299, A549 or HepG2 cells (Supplementary Figures S3A–D).

We therefore investigated whether NRP2 plays a role in p53-R273H- DLX2-mediated induction of scattering cell growth and cell mobility. First, we examined the effects of p53-R273H on NRP2 expression. Again, expression of p53-R273H in H1299 cells downregulated DLX2 protein expression, concomitant with upregulation of NRP2. Further analyses showed that p53-R273H significantly stimulated NRP2 transcription, which was repressed by expression of DLX2 (Figure 4b). Ectopic expression of DLX2 significantly attenuated p53-R273H induced upregulation of NRP2 (Figure 4c).

Second, since NRP2 has been reported promoting cell mobility,^29^ we therefore investigated the effects of p53-R273H-NRP2 axis on expression of several genes involved in epithelial-mesenchymal transition. As shown in Figure 4d, expression of p53-R273H in H1299 cells upregulated NRP2, concomitant with increased regulation of Snail, N-cadherin and Vimentin, which was significantly suppressed by knocked-down of NRP2 using two different NRP2 shRNAs.

Since E-cadherin expression is very low and barely detectable in H1299 cells, we employed Q-PCR analyses to examine the effects of p53-R273H on E-cadherin expression. As shown in Figure 4e, p53-R273H upregulated both NRP2 and Snail expression concomitant with significantly reduced E-cadherin expression, which were completely rescued by knockdown of NRP2. Furthermore, knockdown of NRP2 effectively inhibited p53-R273H-induced scattering cell growth (Supplementary Figure S4), cell migration and invasion (Figures 4f and g). Taken together, these results indicate that NRP2 plays a critical role in p53-R273H-induced cell mobility.

### Ablation of endogenous p53-R273H reduces cell migration via modulation of DLX2 and NRP2 expression

To determine the role of endogenous mutant p53-R273H in cell mobility, we used human lung adenocarcinoma H1975 and human triple negative breast adenocarcinoma MDA-MB-468 cells, both of which express endogenous p53-R273H, and the protein levels of endogenous mutant p53-R273H are compared to the ectopic expression p53-R273H in H1299 (Supplementary Figure S1D).

**Figure 5:**
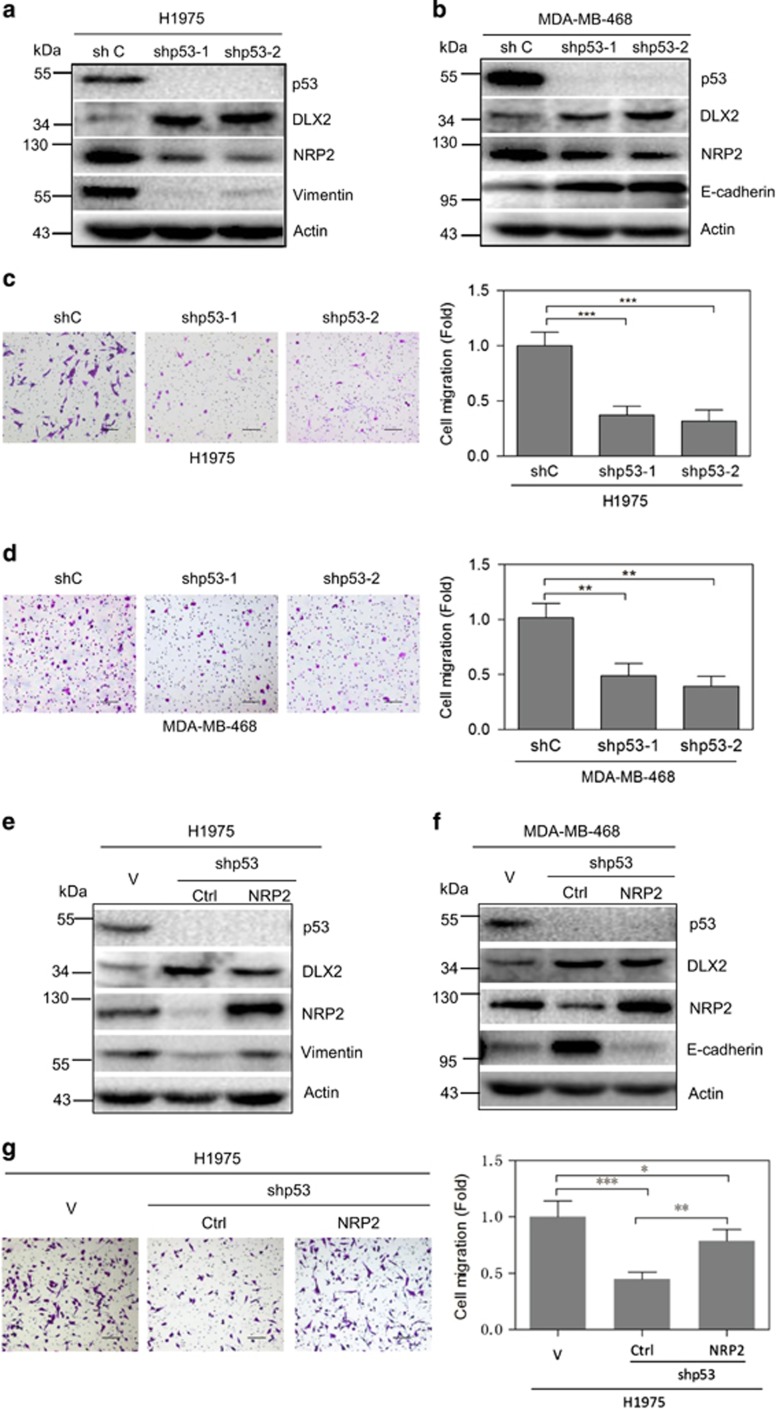

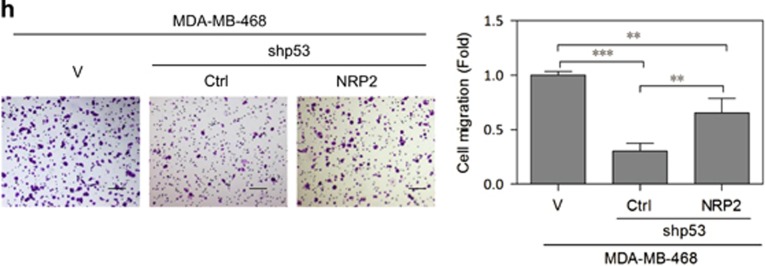
Ablation of endogenous p53-R273H reduces cell migration via modulation of DLX2 and NRP2 expression. (**a–d**) H1975 or MDA-MB-468 cells stably expressing a shRNA specific for p53 (shp53-1 or shp53-2) were subjected to western blot analyses (**a**,**b**) and to transwell migration assays (**c**,**d**). (**e**–**h**) H1975 or MDA-MB-468 cells stably expressing a shRNA specific for p53 (shp53-1) were infected with recombinant lentivirus encoding NRP2. Cells were subjected to western blot analyses (**e**,**f**) and to transwell migration assays (**g**,**h**). Results are presented as means and S.E. from three independent experiments in triplicates. Scale bar =100 *μ*m. * indicated *P*<0.05, ** indicated *P*<0.01, *** indicated *P*<0.005

As shown in Figure 5, knockdown of endogenous p53-R273H by two different shRNAs resulted in significant upregulation of DLX2 and downregulation of NRP2 in both cells. Importantly, knockdown of endogenous p53-R273H leads to significant reduction of cell migration.

To demonstrate that reduced NRP2 expression is critical in depletion of p53-R273H-mediated reduction of cell mobility, we performed rescuing experiments. Ectopic expression of NRP2 significantly, but not completely, reversed migration in both H1975 and MDA-MB-468 cells expressing shRNA specific for p53 (Figures 5e–h).

We then examined whether p53-R273H-NRP2 axis can impact cell proliferation. Our data showed that knockdown of p53-R273H reduced cell proliferation as accessed by 3-(4,5-dimethylthiazol-2-yl)-5-(3-carboxymethoxyphenyl)-2-(4-sulfophenyl)-2H-tetrazolium) assays. However, restoration of NRP2 was unable to restore cell growth of both H1975 and MDA-MB-468 cells (Figures 5e and f; Supplementary Figures S5A and B). Thus, these data indicate that p53-R273H regulates NRP2 to primarily impact on cell mobility.

### Reduced expression of NRP2 inhibits p53-R273H-induced tumor metastasis *in vivo*

**Figure 6:**
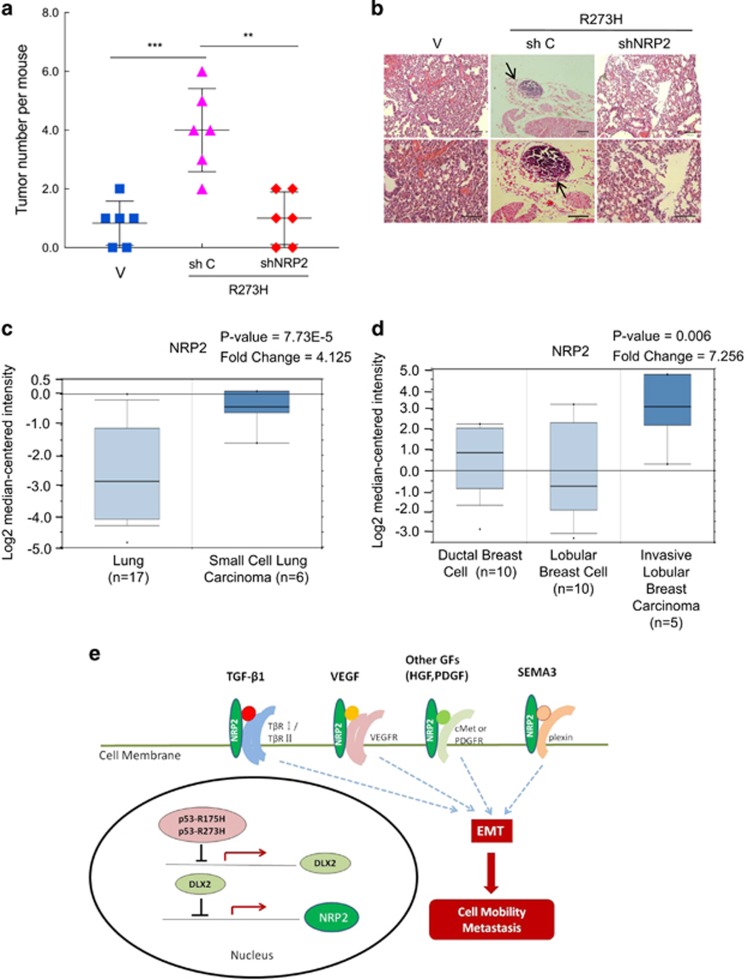
NRP2 is critical in p53-R273H-induced tumor metastasis *in vivo* and is upregulated in human clinical cancer samples. (**a**,**b**) Stable H1299 cells expressing p53-R273H were infected with recombinant lentivirus encoding an shRNA specific for NRP2 (shNRP2-1) or shGFP were tail-vain injected into female nude mice (6 mice per group). Mice were observed daily and killed after 60 days. Lungs were dissected and fixed, and were inspected for metastatic nodules on their surface. The graph represents the number of metastatic nodules in the lung from each mouse. A horizontal line indicates the mean of each group (**a**). Lungs were fixed, embedded in paraffin, sectioned, and stained by H&E for histological analysis (**b**). Metastatic nodules are indicated by arrows. Scale bars=100 *μ*m. ** indicated *P*<0.01, *** indicated *P*<0.005. (**c**,**d**) NRP2 gene expression from Radvanyi Breast and Lung data set. Oncomine (Compendia Bioscience, Ann Arbor, MI, USA) was used for analysis and visualization. (**c**) Box-and-whisker plots representing NRP2 expression in human lung cancer samples. NRP2 fold change: 4.125; *P*=7.73E-5; (**d**) Box-and-whisker plots representing NRP2 expression in human breast cancer samples. NRP2 fold change: 7.256; *P*=0.006. (**e**) A working model in that mutant p53 R273H and NRP2 function in cell motility and tumor metastasis. H&E, hematoxylin and eosin

To determine the role of NRP2 in p53-R273H-induced tumor metastasis *in vivo*, we used stable H1299 cells expressing p53-R273H, or simultaneously expressing p53-R273H and shNRP2 in a mouse lung metastasis model. As shown in Figure 6a, p53-R273H promoted lung metastasis, as evidenced by increased tumor nodules on the lung surface, whereas simultaneous expression of shNRP2 effectively inhibited p53-R273H-induced metastasis. These results were further supported by pathological examinations using hematoxylin and eosin staining (Figure 6b). These results demonstrate that NRP2 is critical in p53-R273H-induced tumor metastasis *in vivo*.

To further explore clinical relevance of NRP2 expression in human cancer specimen, we analyzed Oncomine Database. The data showed that upregulation of NRP2 significantly correlated in human lung cancer and breast cancer specimens (Figures 6c and d).

Taken together, our study reveals a new pathway with which mutant p53-R273H suppresses DLX2 transcription, resulting in downregulation of NRP2 expression, which in turn leads to attenuation of signaling transduction pathway involved in TGFβ, SEMA3 and vascular endothelial growth factor in epithelial-mesenchymal transition, cell mobility and cancer metastasis (Figure 6e).

## Discussion

It is well documented that more than 50% of human tumors/cancers harbor point mutations on p53 gene. In particular, hotspot mutations, including R175H, R248W and R273H, are closely associated with human tumorigenesis. While these mutations have lost their transcription activity in regulation of genes involved in cell cycle progression and apoptosis, it is now clear that these mutations have exhibited gain of functions associated with cancer development, most strikingly, with cancer metastasis. In this study, we demonstrate that all three p53 hotspot mutations, R175H, R248W and R273H, can increase cell mobility with characteristics of scattering cell growth, spindle-like cell morphology and increase cell migration/invasion. We further show that p53-R273H downregulates expression of DLX2, resulting in upregulation of NRP2. In addition, we show that NRP2 plays a critical role in p53-R273H-induced cell mobility *in vitro* and tumor metastasis *in vivo*. Notably, although the levels of ectopically expressed p53-R273H in stable H1299-R273H cells are moderately higher than the endogenous p53-R273H protein levels in H1975 and MDA-MB-468 cells (Supplementary Figure 1D), the biological effects of the ectopically expressed p53-R273H in impacting expression of DLX2 and NRP2 is consistent with the function of endogenous p53-R273H.

Most p53 hotspot mutations are missense mutations located in exons 4–9 encoding the DNA binding domain. These mutations can be divided into DNA contact mutations directly involved in DNA binding, such as R273H and R248W,^25^ and conformational mutations, such as R175H, that alter global conformation of the protein. In our study, either DNA contact mutation (R273H/R248W) or conformational mutation (R175H) can enhance cell mobility, raising an interesting possibility that hotspot p53 mutations share a common gain of function in promoting cancer metastasis.

Notably, restorations of NRP2 can significantly, but not completely, restore cell mobility affected by ablation of endogenous p53-R273H, suggesting that there are alternative targets that contribute to p53-R273H-mediated cell mobility. Keep with this notion, we also found that restoration of NRP2 is unable to rescue cell grow defects coursed by ablation of endogenous p53-R273H. It has been reported that p53-R175H facilitates integrin recycling to promote cell invasion.^8^ p53-R175H can activate EGFR/PI3K/AKT pathway to promote migration and invasion.^30^ p53-R175H can also elevate Twist1 expression to induce epithelial-mesenchymal transition-like transition.^31^ In addition, it has been shown that p53-R273H promotes sustained EGF-induced ERK activation via the miR-27a/EGFR axis to facilitate cell proliferation and tumorigenesis.^32^ Furthermore, p53-R273H can upregulate CXC chemokines and enhances cell migration.^33^ Our study clearly demonstrates that p53-R273H is a gain of function mutant in upregulating NRP2, thereby promoting cell mobility and tumor metastasis.

The DLX2 gene encodes a homeobox transcription important for morphogenesis and development. Deregulation of DLX2 gene expression has been found in human solid tumors and hematologic malignancies.^19, 20^ DLX2^−/−^ mice exhibit defective cell migration, resulting in increased neurite length.^13, 34^ It has reported that p53-R175H can downregulate DLX2 in H1299 cells, although the detail mechanism remains unknown.^27^ Additionally, DLX2 has been implicated in ATM-p53 signaling by modulating protein components of the TTI1/TTI2/TEL2 complex.^35^ In this study, we clearly demonstrate at the molecule level that p53-R273H-mediated downregulation of DLX2 significantly contributes to increased cell migration and invasion.

Multiple reports have indicated a role of NRP2 in cancer metastasis.^36^ For instance, NRP2 is shown to promote metastasis in melanoma^37^ and to enhance migration of hepatocellular carcinoma cells.^29^ In addition, NRP2 promotes metastasis in oesophageal squamous cell carcinoma through deregulation of ERK-MAPK-ETV4-MMP-E-cadherin.^38^ Notably, knockdown of wild-type p53 in colorectal cancer LS174T cells reduces NRP2 expression and tumor vessel information and cell growth.^39^ In this study, we found that p53-R273H suppresses DLX2, resulting in upregulation of NRP2, consistent with a previous study showing that DLX2 directly inhibits NRP2 transcription.^40^ Interestingly, DLX1 can also transcriptionally suppress NRP2.^40^ Whether mutant p53 also regulates NRP2 through DLX1 needs further investigation.

Mutant p53 proteins execute gain of function through multiple mechanisms, at both transcriptional and non-transcriptional levels. For instance, mutant p53 can directly bind to regulatory regions of target genes, such as MDR-1,^41, 42^ proliferating cell nuclear antigen^43^ or vascular endothelial growth factor.^44^ Mutant p53 can also physically interact with other p53 family members, such as p63 and p73, and inhibit transactivation of their respective target genes.^45, 46, 47, 48^ In addition, mutant p53 protein can modulate gene expression through interaction with cellular transcription factors, such as NF-Y,^49, 50^ SREBP2,^51^ Sp1,^52, 53, 54, 55^ ETS1,^56, 57^ and VDR.^58^ On the other hand, mutant p53 protein can interact with other cellular proteins, which are not transcription factors, such as TopB1,^50^ Pin1,^12^ MRE11,^59^ or PML^60^ to modulate various biological processes. The precise mechanism with which p53-R273H suppresses DLX2 transcription must wait for further investigation.

Together, our study provides additional evidence that mutant p53-R273H is a gain of function mutant that promotes cell migration, invasion and tumor metastasis. This study reveals that p53-R273H-DLX2-NRP2 axis may play an important patho-physiological role in tumor development.

## Materials and methods

### Cell culture, generation of stable cell lines and viral infections

Human NSCLC H1299, H1975 and A549, human hepatocellular carcinoma HepG2 and human triple negative breast cancer MDA-MB-468 cells were obtained from ATCC. Human colon cancer HCT116-p53^−/−^ cell line was a generous gift from Dr. Bert Vogelstein. H1975 cells were maintained in RPMI-1640 media and other cells were maintained in Dulbecco’s modified Eagle’s medium (DMEM), supplemented with 10% fetal bovine serum (Hyclone Inc, Logan, UT, USA), 1% L-glutamine, 100U penicillin–streptomycin at 37 °C in a humidified incubator under 5% CO_2_.

For the generation of stable cell lines, H1299 and HCT116-p53^−/−^ cells were transfected with either pCMV-neo-Bam-p53-R273H, pCMV-neo-Bam-p53-R175H, or pCMV-neo-Bam-p53-R248W by Lipofectamine 2000 (Invitrogen Inc, Hampshire, England). Twenty-four hours after transfection, cells were selected using fresh growth medium containing 1000 *μ*g/ml G418 (sigma Inc, Louisiana, USA).

To generate recombinant lentiviruses expressing pLVX-Puro-DLX2, pLenti-M3-Blasticidin-NRP2, pLKO.1-Puro-shp53, pLKO.1-Puro-shDLX2, pLKO.1-Puro-shNRP2 or pLKO.1-Puro-shGFP, which was used to co-transfected HEK-293T cells with psPAX2 and pMD2G packaging plasmids using Lipofectamine 2000 (Invitrogen Inc). Supernatants containing recombinant lentiviruses were used in subsequent viral-infection experiments. Pool of drug-resistant cells stably expressing the desired gene or shRNA was selected against either puromycin or blasticidin, respectively.

### Expression constructs

p53-R273H, p53-R175H, p53-R248W plasmids were gifts from Dr. Bert Vogelstein. Human DLX2 was cloned by PCR amplification and sub-cloned into pLVX-Puro. Human NRP2 gene was purchased from Vigene Biosciences Inc (Shangdong, China) and sub-cloned in pLenti-M3-Blasticidin. All constructs used in this study were confirmed by DNA sequencing. Specific shRNA sequences are listed below:

p53: shRNA-1: 5′-AAG-ACTCCAGTGGTAATCTACT-3′

shRNA-2: 5′-CACCATCCACTACAACTACAT-3′

DLX2: shRNA-1: 5′-GCACCATCT- ACTCCAGTTT-3′

shRNA-2: 5′-AGAGACCACTTATCCTCATTGCTTA-3′

NRP2: shRNA-1: 5′-AGATTGTCCTCAACTTCAA-3′

shRNA-2: 5′-ACACGACTGCAAGTATGAC-3′

### Western blot analyses

Cells were lysed in EBC_250_ lysis buffer. (250 mM NaCl, 25 mM Tris, pH 7.4, 0.5% Nonidet P-40, 50 mM NaF, 0.5 mM Na_3_VO_4_, 0.2 mM phenylmethylsulfonyl fluoride, 20 *μ*g/ml aprotinin and 10 *μ*g/ml leupeptin). Equal amounts of total proteins were separated by SDS-PAGE, transferred to PVDF membrane and hybridized to an appropriate primary antibody and horseradish peroxidase-conjugated secondary antibody for subsequent detection by enhanced chemiluminescence.

Antibody used in this study were as follows: p53 (DO-1; 1:2000 dilution; Cat # sc-126; Santa Cruz Inc, CA, USA), NRP2 (1:200; Cat#Ab155680; Abcam, Cambridge, MA, USA), N-cadherin (1:1000; Cat#2447-1; Abcam, Cambridge, MA, USA), Vimentin (C-20, 1:1000, Cat#sc-7557; Santa Cruz Inc, CA, USA), Snail (1:1000; 3895, Cat#, Cell Signaling, MA, USA), Actin (C-11; 1:2000; Santa Cruz), GAPDH (1:2000; Cell Signaling, MA, USA) and DLX2 (1:200; generated from Zhengneng Biotechnology, Chengdu, China).

### Quantitative RT-PCR

For quantitative RT-PCR (Q-PCR) analyses, total RNAs were isolated from cells using an RNA extraction kit (Qiagen, Hilden, Germany) according to the manufacturer’s instruction. Complementary DNAs (cDNA) were generated using the first-strand cDNA kit (Invitrogen Inc). Q-PCR analyses were performed in CFX96 Real-Time System (Bio-Rad, Hercules, CA, USA) using SYBR Green Master Mix (Bio-Rad) with the following PCR conditions: 95 °C annealing 2 min, 30–40 cycles of 30 s at 95 °C, 30 s at 55 °C and 1 min at 72 °C, followed by a 10 min 72 °C incubation. After finishing these cycles, a melting curve was generated to verify specificity. GAPDH was used as a reference. DNA sequences of the Q-PCR primers are list below:

GAPDH fw 5′-TGGACTCCACGACGTACTCA-3′

GAPDH rev 5′-AATCCCATCACCATCTTCCA-3′

p53 fw 5′-CCTCACCATCATCACACTGG-3′

p53 rev 5′-GCTCTCGGAACATCTCGAAG-3′

DLX2 fw 5′- GCACATGGGTTCCTACCAGT-3′

DLX2 rev 5′-ACTTTCTTTGGCTTCCCGTT-3′

NRP2 fw 5′-GGATGGCATTCCACATGTTG-3′

NRP2 rev 5′- TGTGAAAGGTCAGGGAGAGGAT-3′.

### Colony formation assay

H1299 cells were seeded at single cell density in six-well plates and were allowed to grow in a humidified incubator under 5% CO_2_ for 5–10 days. Colonies were fixed for 20 min with methanol and were stained for 30 min with crystal violet (0.4% in methanol). Cell morphology was captured using a phase-contrast microscope.

### Wound healing assay

To examine cell migration, live-cell imaging was performed on a Leica AF6000 inverted microscope at 37 °C under 5% CO_2_. H1299 cells were seeded in six-well plates and grown to 100% confluence. A ‘wound’ was created using pipette tips. Cells were washed with 1 × PBS and maintained in DMEM with 1% FBS. The cell images at exact same place were recorded every 30 min using a time-lapse microscopy (Leica AF6000).

### Transwell assays for cell migration and invasion

Transwell assays were performed as described.^61^ Briefly, cell migration was measured using 6.5 mm, 8.0 *μ*m-pore polycarbonate membrane transwell inserts (BD Biosciences, San Jose, CA, USA). Cell invasion was measured using matrigel-coated inserts (BD Biosciences, San Jose, CA, USA). Cells (5.0 × 10^4^ or 3 × 10^5^ for MDA-MB-468) were suspended in serum-free DMEM media and seeded into the inner chamber. The outer chamber was filled with normal growth media. Cells were incubated for 12–24 h. Non-migrating cells were carefully removed with a cotton swab. Migrating cells were stained with 0.4% crystal violet in methanol for 10–20 min at room temperature, and photographed under a Zeiss light microscope. At least 100 cells in total from five random fields were counted.

### *In vivo* metastasis assays

2.0 × 10^6^ stable H1299 cells [p53-R273H (R273H), (R273H +shNRP2) or (R273H+shC)] in 100 *μ*l PBS were injected into the lateral tail vein of 6-week-old female BALB/c nude mice (DaShuo Biotechnology, Chengdu, China). Mice were observed daily and killed after 60 days. The lungs were dissected and observed for metastatic nodules. Lungs were fixed overnight in 4% formaldehyde, embedded in paraffin and sectioned. Lung sections were stained using hematoxylin and eosin.

### Luciferase assay

H1299 cells grown in 24-well plates were co-transfected with plasmids expressing wild-type p53 or p53-R273H in the presence of DLX2-luciferase and pRL-TK-Renilla luciferase reporter constructs. Twenty-four hours after transfection, luciferase activity was measured using a Dual Luciferase Reagent (Promega, Madison, WI, USA) according to the manufacturer’s instructions. Luciferase activity was normalized to Renilla luciferase activity.

### Oncomine analyses and statistical analyses

Oncomine database (Compendia Bioscience, Ann Arbor, MI, USA; https://www.Oncom ine.org/resource/login.html) was used for analyses of gene expression. Quantitative data were analyzed statistically using Student’s *t*-test to assess significance. Data are presented as means±s.e., as noted on figure legends.

### 3-(4,5-dimethylthiazol-2-yl)-5-(3-carboxymethoxyphenyl)-2-(4-sulfophenyl)-2H-tetrazolium) Assay

To examine cell growth/viability, we used 3-(4,5-dimethylthiazol-2-yl)-5-(3-carboxymethoxyphenyl)-2-(4-sulfophenyl)-2H-tetrazolium) assay according to the manufacturer’s instruction. Briefly, 5000 H1975, or MDA-MB-468 cells per well were seeded in triplicates in a 96 well-plate, and incubated at 37 °C under 5% CO_2_. Ten *μ*l of Dye solution (Promega) was added to each well at indicated time intervals (0, 24, 48, 72 and 96 h) after seeding.^62^ Absorbance at 490 nm was recorded using a 96-well plate reader (Thermo Scientific Varioskan flash).
