# Supplementary figures and images for: p53-R273H upregulates neuropilin-2 to promote cell mobility and tumor metastasis
Source: Cell Death Dis. 2017 Aug 10;8(8):e2995–. doi: 10.1038/cddis.2017.376 (PMC5596564; doi:10.1038/cddis.2017.376)

A

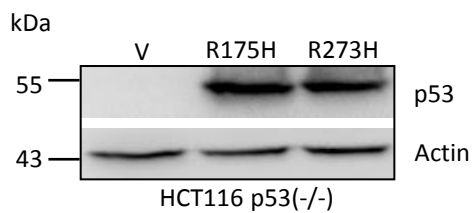

B

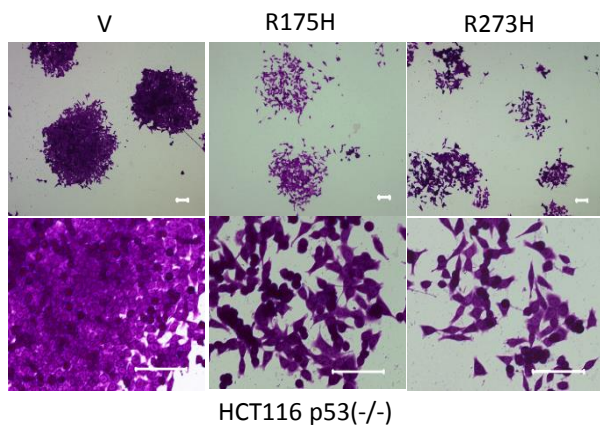

C

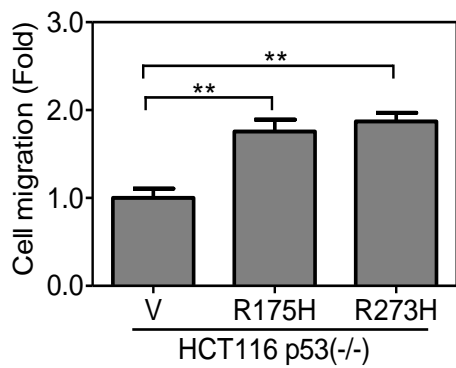

D

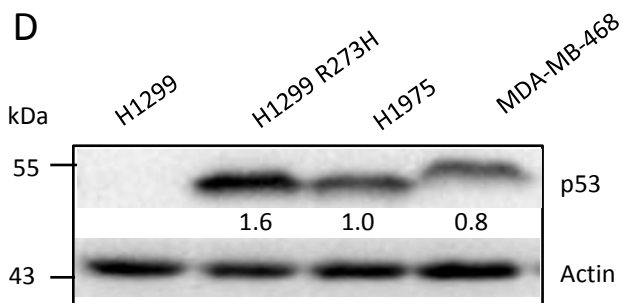

A

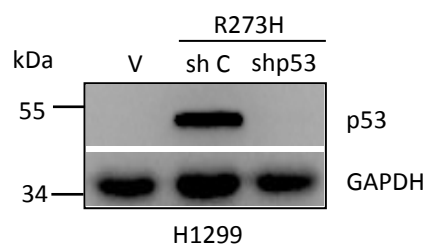

B

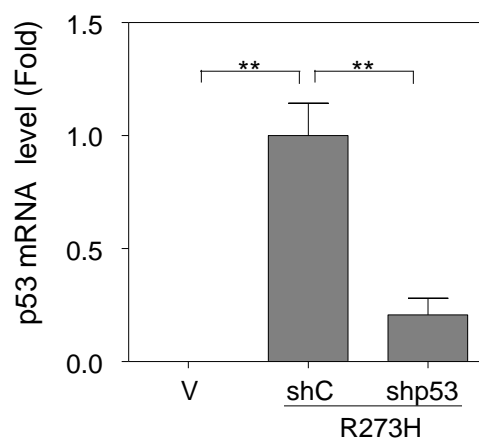

C

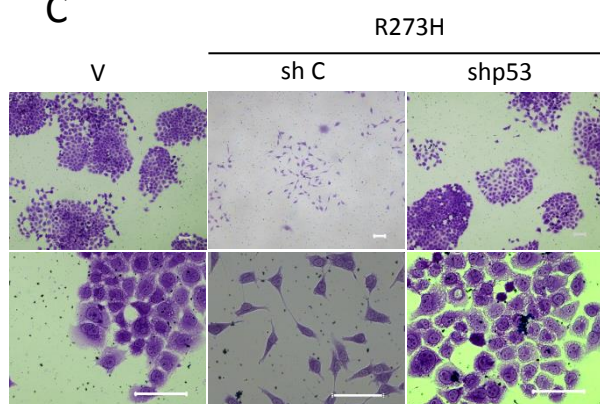

D

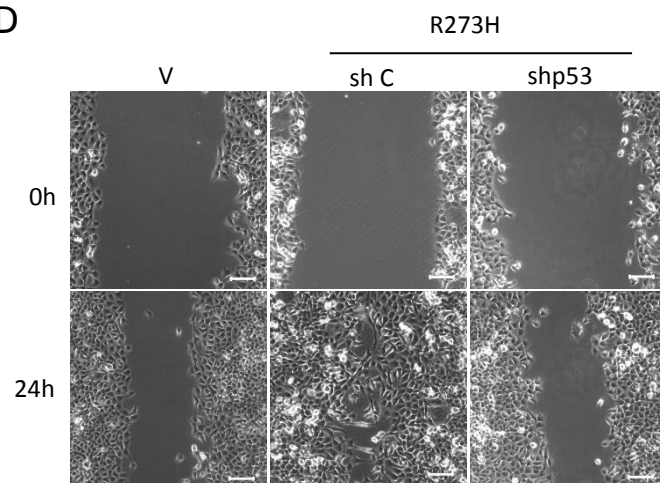

E

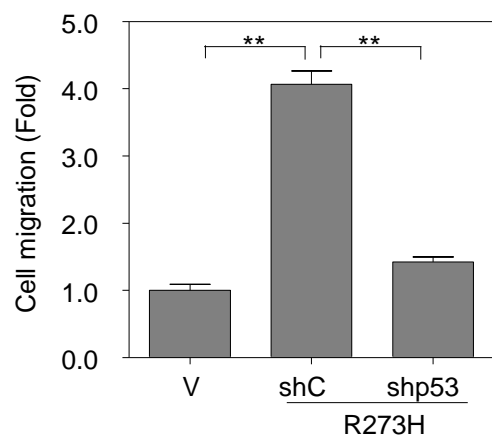

F

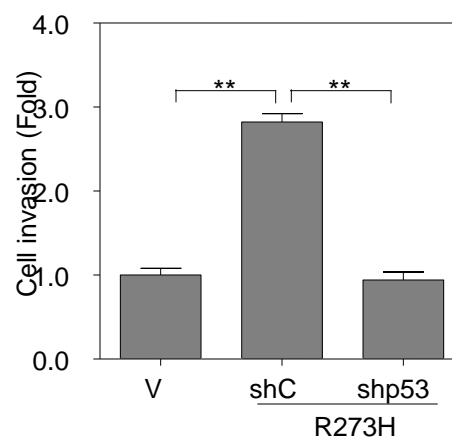

**A**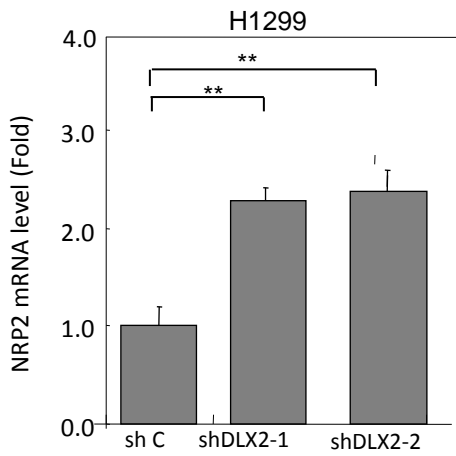**B**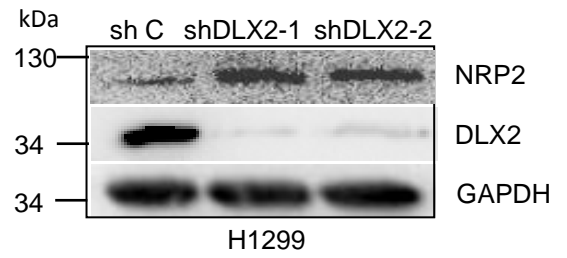**C**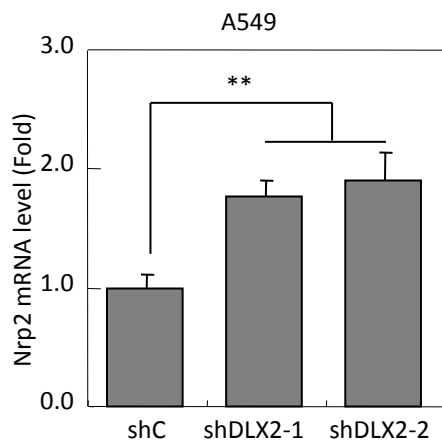**D**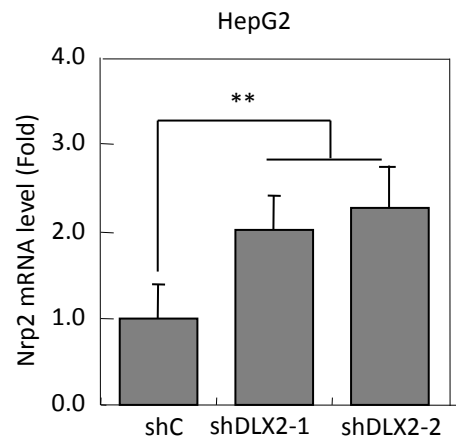

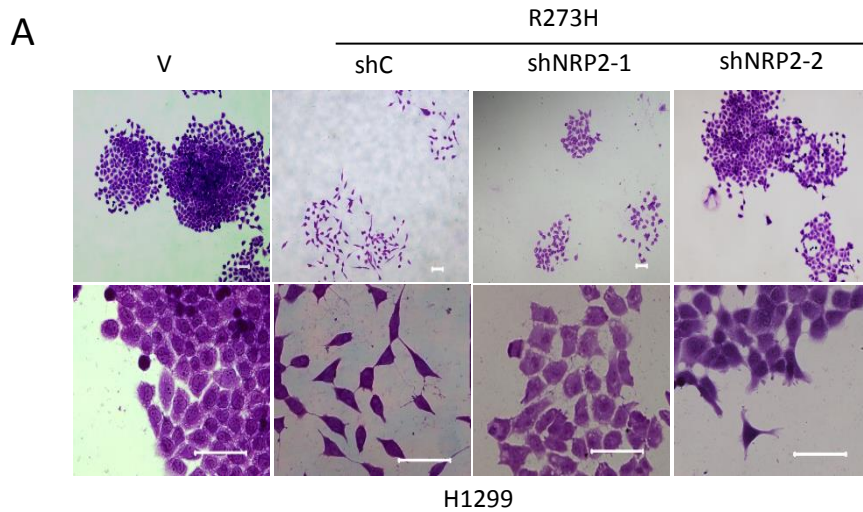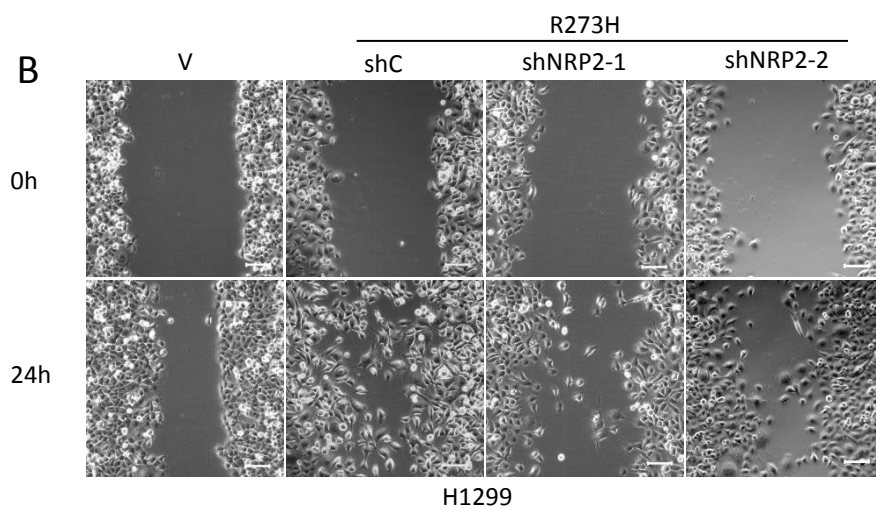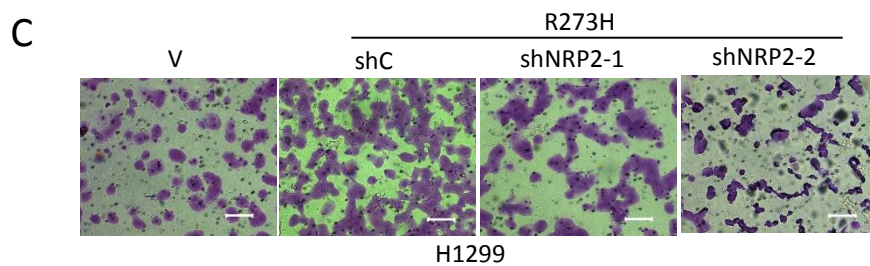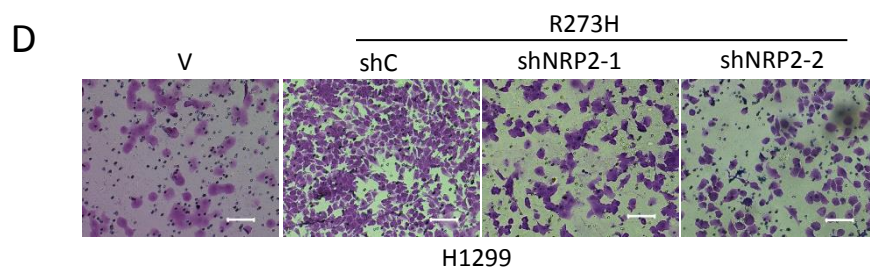

A

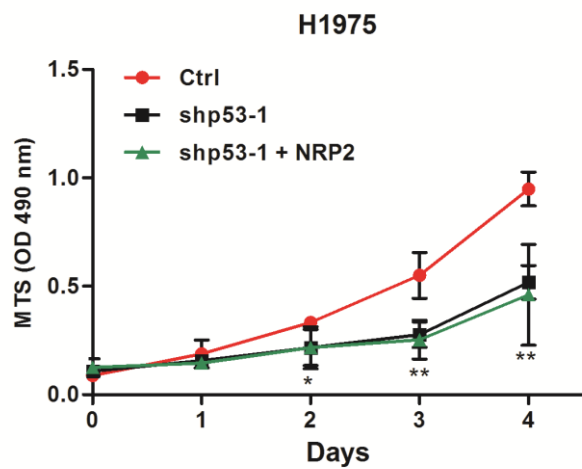

B

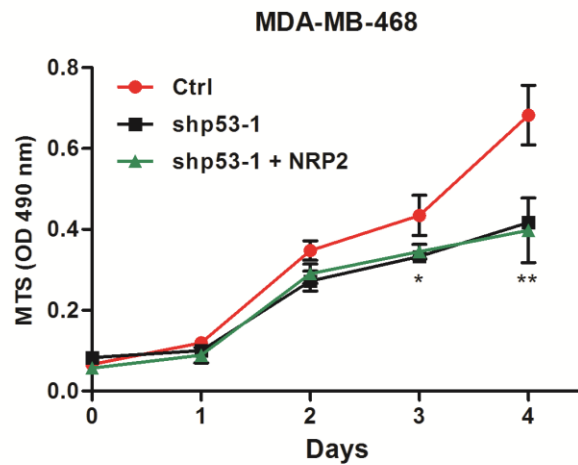

Supplement: Supplementary Figures [file cddis2017376x1.pdf]
