# Supplementary material for: p53-R273H upregulates neuropilin-2 to promote cell mobility and tumor metastasis
Source: Cell Death Dis. 2017 Aug 10;8(8):e2995–. doi: 10.1038/cddis.2017.376 (PMC5596564; doi:10.1038/cddis.2017.376)
Supplement: Supplementary Figure Legends [file cddis2017376x2.docx]

**Supplemental Figure Legends**

**Figure S1. Expression of mutant p53 in HCT 116 p53 -/- cells promotes cell scattering, migration and invasion.**

(A-C) p53-R173H (R175H), p53-R273H (R273H) or Vector control (V), were stably expressed in HCT116 p53-/- cells. Whole-cell lysates were subjected to western blotting (A) for p53 expression using p53 (DO-1) antibody. Actin was used as a loading control. Cells were plated at single-cell density and cultured for 5 days. Colonies were then fixed and stained with crystal violet and were photographed (B). Scale bar =100 µm. Cells were subjected to cell migration using transwell assays. Results are presented as means and SE from independent experiments in triplicates. Scale bar =100 µm. ** indicated p<0.01 (C).

(D) Cell lysates from H1975 and MDA-MB-468 cells expressing endogenously p53-R273H were subjected for western blotting for p53 expression. Cell lysates from parental H1299 or stable H1299 cells expressing p53-R273H (H1299-R273H) were used in parallel. p53 protein levels were subjected to qualification analyses using actin for normalization.

.

**Figure S2. p53**-**R273H in promotes H1299 cells scattering, migration and invasion**

(A-F) H1299 cells stably expressing p53-R273H were infected with recombinant lentivirus expressing shRNA against p53 (shp53) or a control green fluorescent protein (shC) ^[8](#_ENREF_8)^. (A) Whole-cell lysates were subjected to western blotting for p53. (B) Q-PCR analyses were performed for p53 expression. GAPDH was used as control.

(C) Cells were plated at single-cell density and cultured for 5 days. Colonies were then fixed and stained with crystal violet and were photographed. Cells were subjected to wound-healing assay（D）, cell migration (E) or invasion assays (F) using transwell systems. Results are presented as means and SE from independent experiments in triplicates. Scale bar =100 µm. ** indicated p<0.01.

**Figure S3. Knock down of DLX2 up regulates NRP2 expression.**

(A-B) H1299 cells stably expressing one of two different shRNA against DLX2 (shDLX2-1 or shDLX2-2) or a control shRNA against green fluorescent protein [^8^](#_ENREF_8) were subjected to Q-PCR analyses for NRP2 expression (A). Cells were subjected to western blotting analyses for DLX2 and NRP2 expression (B). Results are presented as means and SE from three independent experiments performed in triplicates. ** indicated p<0.01.

(C-D) A549 or HepG2 cells stably expressing one of two different shRNA against DLX2 (shDLX2-1 or shDLX2-2) were subjected to Q-PCR analyses for NRP2 expression. Results are presented as means and SE from three independent experiments performed in triplicates. ** indicated p<0.01.

**Figure S4.** **p53**-**R273H up-regulates NRP2 to promote cell scattering growth, migration and invasion.**

(A-D) Stable H1299-(p53-R273H) cells expressing shRNA specific for NRP2 (shNRP2-1, shNRP2-2) or control cells (H1299-V) were plated at single-cell density and cultured for 5 days. Colonies were then fixed and stained with crystal violet and were photographed (A). Cells were subjected to wound-healing assay (B), cell migration (C) or invasion assays (D) using transwell systems. Scale bar =100 µm. Results performed three independent experiments in triplicates.

**Figure S5. Knockdown of endogenous p53-R273H reduces cell proliferation.**

(A-B) H1975 or MDA-MB-468 cells stably expressing shp53-1 were infected with recombinant lentivirus encoding NRP2. Cells were subjected to cell proliferation analysis by MTS assays. Results are presented as means and SE from three independent experiments in triplicates. * indicated p<0.05, ** indicated p<0.01.
